# C‐Cbl regulates c‐MPL receptor trafficking and its internalization

**DOI:** 10.1111/jcmm.15785

**Published:** 2020-09-20

**Authors:** Melanie Märklin, Claudia Tandler, Hans‐Georg Kopp, Kyle L. Hoehn, Leticia Quintanilla‐Martinez, Oliver Borst, Martin R. Müller, Sebastian J. Saur

**Affiliations:** ^1^ Clinical Collaboration Unit Translational Immunology German Cancer Consortium (DKTK) German Cancer Research Centre (DKFZ) University Hospital Tübingen Tübingen Germany; ^2^ Department of Molecular Oncology and Thoracic Oncology Robert‐Bosch‐Hospital Stuttgart Stuttgart Germany; ^3^ School of Biotechnology and Biomolecular Sciences University of New South Wales Sydney NSW Australia; ^4^ Department of Pathology University of Tübingen Tübingen Germany; ^5^ Department of Kardiology and Angiology University Hospital Tübingen Tübingen Germany; ^6^ Department of Hematology, Oncology, Clinical Immunology and Rheumatology University Hospital Tübingen Tübingen Germany; ^7^ Department of Hematology, Oncology and Immunology Klinikum Region Hannover KRH Klinikum Siloah Hannover Germany

**Keywords:** C‐Cbl, c‐MPL, megakaryocytes, platelets, thrombocytosis

## Abstract

Thrombocyte formation from megakaryocyte and their progenitor cells is tightly regulated by thrombopoietin (TPO) and its receptor c‐MPL, thereby maintaining physiological functionality and numbers of circulating platelets. In patients, dysfunction of this regulation could cause thrombocytopenia or myeloproliferative syndromes. Since regulation of this pathway is still not completely understood, we investigated the role of the ubiquitin ligase c‐Cbl which was previously shown to negatively regulated c‐MPL signalling. We developed a new conditional mouse model using c‐Cbl^fl/fl^Pf4^Cre^ mice and demonstrated that platelet‐specific knockout of c‐Cbl led to severe microthrombocytosis and impaired uptake of TPO and c‐MPL receptor internalization. Furthermore, we characterized a constitutive STAT5 activation c‐Cbl KO platelets. This study identified c‐Cbl as a potential player in causing megakaryocytic and thrombocytic disorders.

## 1 INTRODUCTION

During hematopoiesis, the development of bone marrow progenitor cells into mature blood cells and maintenance of physiological numbers of circulating blood cells are tightly regulated by several cytokines and growth factors. In this context, thrombopoietin (TPO) and its corresponding receptor c‐MPL play a central role during thrombopoiesis, the developmental process of thrombocyte formation from megakaryocyte (MK) and their progenitor cells.^1^, ^2^

Disruption of TPO signalling results in thrombocytopenia, reduction of hematopoietic stem cells and can even lead to a state of complete bone marrow failure, called aplastic anaemia.^3^, ^4^, ^5^ In contrast, uncontrolled TPO signalling due to, for example, mutations in c‐MPL or its downstream signalling proteins results in a hyperproliferative phenotype, which causes myeloproliferative syndromes.^6^, ^7^, ^8^ Therefore, a tight control of TPO‐mediated signalling is necessary to maintain physiological hematopoiesis.

The proto‐oncogene c‐CBL (casitas B cell lymphoma) is involved in ubiquitination of the TPO receptor c‐MPL and thereby negatively regulating TPO signalling.^9^ C‐Cbl is expressed in hematopoietic cells and MKs and is part of the Cbl protein family, which carry evolutionary conserved RING finger domains and show E3 ubiquitin ligase activity. Ubiquitination of target proteins by c‐Cbl is induced by its phosphorylation in response to TPO stimulation.^10^, ^11^, ^12^, ^13^, ^14^

In case of losing the negative regulation of the TPO signalling, c‐Cbl null mice show a clear expansion in hematopoietic stem/progenitor cell numbers as well as being hyperproliferative and sensitive to TPO stimulation which induces STAT5 signalling.^15^ In patients with myeloproliferative disorders such as essential thrombocythemia (ET), studies have shown an impaired platelet‐dependent TPO clearance, suggesting that thrombocytosis in ET may be attributed to an alteration in TPO signalling via its receptor c‐MPL.^16^

Here, we report on a new genetic model to specifically delete c‐Cbl in the MK lineage by using a conditional knockout strain with a platelet and MK specific Pf4^cre^. C‐Cbl^fl/fl^Pf4^Cre^ mice present with a microthrombocytosis and an accelerated platelet turnover along with faster platelet recovery after platelet depletion. The number of MKs was elevated along with the number of their progenitor cells in the bone morrow, without signs of myelofibrosis. Interestingly, there was no difference in spleen size compared to wild‐type (WT) mice. TPO receptor c‐MPL surface expression was reduced on platelets of c‐Cbl^fl/fl^Pf4^Cre^ mice while showing impaired internalization and markedly reduced ability to take up TPO, resulting in elevated TPO serum levels in these mice.

## 2 MATERIALS AND METHODS

### 2.1 Mice

Mice bearing a conditional floxed allele of c‐Cbl (Cbl^fl/fl^) C57BL/6‐Cbl^tm1Dejs^ (Australian Phenomics Facility) were crossed with Pf4^Cre^ mice were obtained from Jackson Laboratory (stock #008535) to obtain c‐Cbl^fl/fl^Pf4^Cre^. Homozygous c‐Cbl^fl/fl^Pf4^Cre^ (KO) mice with megakaryocytic deletion of c‐Cbl were used as the experimental cohort, while c‐Cbl^fl/fl^ (WT) mice without c‐Cbl deletion served as controls. All mice were age‐ and sex‐matched and were killed at the indicated time‐points by overdosing inhalation anaesthesia. Mice were maintained under specific pathogen‐free conditions. All animal experiments were performed with the authorization of the Institutional Animal Care and Use Committee of the University of Tübingen according to German federal and state regulations.

### 2.2 Western blot

Protein lysates were isolated from purified B cells, platelets or in vitro generated MKs with NP‐40 buffer (Thermo Scientific) containing 1 mmol/L PMSF (Sigma‐Aldrich) and PI cocktail (Thermo Scientific) for 30 minutes on ice followed by 10 minutes centrifugation at 15 000 *g*. The supernatant was used for SDS‐PAGE followed by Western blot analysis. Antibodies for c‐CBL (#2747, 1:1000), Actin (8H10D10, 1:5000) (all from Cell Signaling) and c‐MPL (#06‐944; Merck) and horseradish peroxidase‐coupled goat or swine anti‐rabbit secondary antibody (Southern Biotech) were used.

### 2.3 Flow cytometry

The peripheral blood and flushed bone marrow were lysed with ammonium chloride buffer (0.150 mmol/L NH_4_Cl, 0.1 mmol/L EDTA, 0.150 mmol/L KHCO_3_) to eliminate erythrocytes followed by staining with fluorochrome‐labelled CD19 (eBio1D3), CD3 (145‐2C11) CD5 (53‐7.3), CD4 (GK1.5), CD8 (53‐6.7), Gr‐1 (RB6‐8C5), CD11b (M1/70), Sca‐1 (D7), c‐kit (2B8), CD16/32 (2.4G2), CD34 (RAM34), Nkp46 (29A1.4), CD41 (eBioMWReg30) and/or CD150 (9D1) antibodies (Abs). Dead cells were excluded by propidium iodide (PI, 1:1000; Sigma‐Aldrich) staining, and Lineage (LIN) Cocktail contained biotinylated antibodies for CD11b (M1/70), CD3 (145‐2C11), Ter119 (Ter119) and Gr‐1 (RB6‐8C5) and B220 (RA3‐6B2) and were stained with streptavidin‐PerCP. Cells were analysed with the Canto II cytometer (BD Bioscience). Data were obtained with the BD FACSDivaTM (BD Bioscience) and analysed with the FlowJo software. Exemplary gating strategies are shown in Figure S1.

### 2.4 Phospho‐flow analysis

Platelets and flushed bone marrow were prepared and stained with fluorochrome‐labelled Abs as described above in the Material and Methods section. Cells were stimulated according to the manufacturer's instructions of the PerFix EXPOSE Kit (Beckman Coulter) with 100 ng/mL TPO or PMA (50 ng/mL) + Ionomycin (500 ng/mL) at 37°C for the indicated time‐points. Cells were immediately fixed and permeabilized followed by intracellular staining of ERK1/2 (clone 137F5), P‐ERK1/2(T202/Y204) (clone D13.14.4E) (1:400/1:800) and STAT5 (clone 3H7), P‐STAT5(Y694) (clone D47E7) (1:100/1:200) and respective isotype controls. Detection was performed with an α‐rabbit F(ab')2‐FITC conjugate (1:250) (all from Cell Signaling Technology), and samples were measured with the LSRFortessa cytometer (BD Bioscience).

### 2.5 Differentiation and cultivation of BM megakaryocytes in vitro

Bone marrow of c‐Cbl^fl/fl^ and c‐Cbl^fl/fl^Pf4^Cre^ mice was flushed, and hematopoietic stem cells were cultured for 3 days in MK‐media (Opti‐MEM, 1% penicillin/streptomycin (Gibco) and 25 ng/mL recombinant TPO (PeproTech) at 37°C and 5% CO_2_. On day 3 of culturing, mature MKs were enriched using a BSA density gradient (4% and 2% BSA in PBS; Sigma‐Aldrich) and enriched MKs were washed with PBS and prepared for further analysis.

### 2.6 TPO measurements

Thrombopoietin plasma levels were obtained with citrate plasma of different mice and measured with ELISA (Quantikine; R&D). For detection of the TPO uptake, peripheral blood was washed with Tyrode's buffer + 2% BSA + 2.3 µmol/L PGE and once with PBS. 1 × 10^6^ platelets were resuspended in MK‐media + 2000 pg/mL recombinant TPO and mixed gently by flicking the tube. Platelets were incubated at 37°C and constant shaking for 2 hours followed by TPO ELISA of the supernatant. TPO uptake was calculated as follows “$TPO\;pg/mL\;untreated\;control\;‐\;TPO\;pg/mL\;1\times 10^{6}\;Platelets$”.

### 2.7 Immunohistochemistry

For morphological bone analysis, femurs were fixed in 4% buffered formalin for at least 24 hours. Decalcification was performed in EDTA solution at room temperature for 2‐3 days. The decalcified bones were embedded in paraffin and cut in 3‐μm‐thick sections and stained with haematoxylin and eosin (H&E). Spleens were fixed in 4% formalin‐ and paraffin‐embedded. For histology, 3‐µm‐thick sections were cut and stained with H&E and Gomori stain. Immunohistochemistry was performed on an automated immunostainer (Ventana Medical Systems, Inc) according to the company's protocols for open procedures with slight modifications. All slides were stained with the antibody GPIbα (Emfret Analytics). Appropriate positive and negative controls were used to confirm the adequacy of the staining. MKs were counted at 20× HPF (magnification 200×).

### 2.8 qRT‐PCR

RNA was isolated from in vitro generated MKs and liver pellets using RNeasy plus mini kit (Qiagen) followed by reverse transcription (SuperScript II; Invitrogen). Quantitative PCR was performed using SYBR Select Mastermix (Thermo Fisher Scientific) in a LC480 Lightcycler (Hoffmann‐La Roche). QuantiTect primer assays for TPO (Cat# QT00100457), c‐MPL (Cat# QT00112119), Gapdh (Cat# QT01658692) were purchased from Qiagen, and β‐Actin Fwd 3′‐TCTTGGGTATGTAATCCTGTGGCA‐5′; Rv 3′‐ACTCCTGCTTGCTGATCCACATCT‐5′ were synthesized from Sigma‐Aldrich.

### 2.9 Platelet analysis

Retroorbital blood was collected, and whole blood analysis was performed using an automated Bayer Advia 120 MultiSpecies Analyzer (Bayer HealthCare). For platelet analyses, peripheral blood was collected in an anti‐coagulant mixture containing Aster Jandl (85 mmol/L sodium citrate dehydrate, 69 mmol/L citric acid, 20 mg/mL glucose, pH 4.6) and platelet buffer (10 mmol/L HEPES, 140 mmol/L NaCl, 3 mmol/L KCl, 0.5 mmol/L MgCl_2,_ 0.5 mmol/L NaHCO_3_, 10 mmol/L glucose, pH 7.4). After centrifugation (120 g), platelet‐rich plasma (PRP) was used for further analysis.

Mice were iv injected with 2 µg DyLight‐488‐conjugated anti‐GPIbβ Ig (X488, Emfret Analytics) antibody, and platelets were analysed by flow cytometry for CD41 and GPIbβ co‐expression. Platelet lifespan was assessed by normalizing costained platelets at indicated time‐points to the control measurement 3‐6 hours after Ab injection.

For analysis of platelet turnover, X488‐treated mice were iv injected with 600 µg NHS‐biotin 24 hours later. After 3 hours, PRP was isolated and stained with CD41‐PE and streptavidin‐APC (1:100) followed by flow cytometry.

For determination of reticulated platelets, PRP was stained with thiazole orange (TO) (0.1 µg/mL) and CD41‐PE followed by fixation with 1% PFA and flow cytometry. To analyse the recovery of platelets, mice were injected with 3 µg/g of bodyweight depletion Ab (#R300; Emfret Analytics) i.p. and platelets were analysed at the indicated time‐points.

Analysis of the c‐MPL receptor internalization was performed as follows: PRP was stimulated with 25 ng/mL TPO at 37°C for the indicated time‐points. Platelets were washed with ice‐cold wash buffer (140 mmol/L NaCl, 5 mmol/L KCl, 12 mmol/L sodium citrate, 10 mmol/L glucose, 12.5 mmol/L sucrose, pH 6.0) and stained with CD41‐PE and c‐MPL (AMM2) in platelet buffer for 30 minutes at 4°C followed by staining with anti‐ratIgG_1_‐APC conjugate (30 minutes, 4°C) after washing again with platelet wash buffer. C‐MPL receptor expression was measured in ice‐cold platelet buffer by flow cytometry.

### 2.10 Statistical analysis

For statistical analysis, GraphPad Prism 7.03 (GraphPad Software) was used. Mean values and SEM are shown. Distributions of the values were tested with Shapiro‐Wilk normality test. The 95% confidence level was used, and *P*‐values were calculated with an unpaired two‐tailed Student's *t* test or an unpaired two‐tailed Welch's *t* test in the case of normally distributed data. Significance of not normally distributed data was either calculated with a paired two‐tailed Wilcoxon matched‐pairs signed‐rank test or a two‐tailed unpaired Mann‐Whitney test. An unpaired analysis of variance (ANOVA) was used to analyse the differences among group means. *P*‐value of *P* < .05 (*) was used as cut‐off for significance.

## 3 RESULTS

### 3.1 c‐Cbl knockout leads to microthrombocytosis and lymphocytosis

**FIGURE 1:**
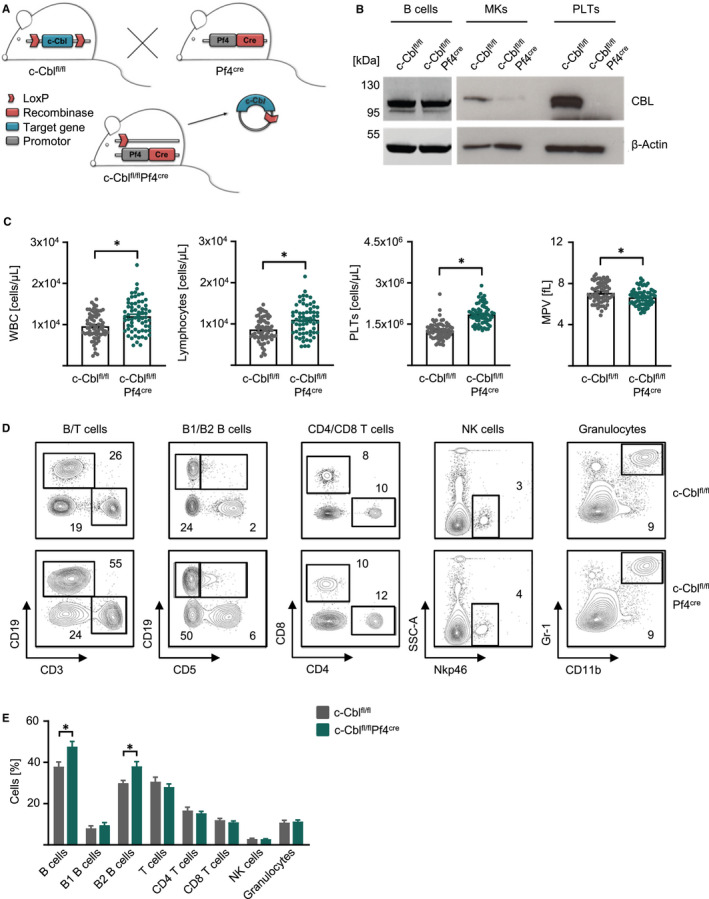
C‐Cbl‐deficient mice showed increased microthrombocytosis and lymphocytosis. A, Mouse breeding scheme to generate c‐Cbl^fl/fl^Pf4^Cre^ mice. B, c‐CBL protein expression in CD19^+^ B cells, megakaryocytes and platelets from c‐Cbl^fl/fl^ and c‐Cbl^fl/fl^Pf4^Cre^ mice assessed by Western blotting. Actin was used as loading control. C, White blood count (WBC), lymphocytes, platelet numbers (PLTs) and mean platelet volume (MPV) (n = 65‐66 per group) were analysed in c‐Cbl^fl/fl^ and c‐Cbl^fl/fl^Pf4^Cre^ mice at an age of 8‐16 wk (Mean ± SEM, *P ≤ .05). D, Representative FACS blots for flow cytometric analysis of B cells (CD19^+^), T (CD3^+^, CD3^+^CD4^+^, CD3^+^CD8^+^) cells, B1 (CD19^+^CD5^+^) and B2 (CD19^+^CD5^−^) B cells, NK cells (Nkp46^+^) and granulocytes (Gr‐1^+^CD11b^+^) in the peripheral blood of c‐Cbl^fl/fl^ and c‐Cbl^fl/fl^Pf4^Cre^ mice at an age of 12‐16 wk. E, Flow cytometric analysis of the peripheral blood of c‐Cbl^fl/fl^ and c‐Cbl^fl/fl^Pf4^Cre^ mice at an age of 12‐16 wk (n = 9 per group, Mean ± SEM, *P ≤ .05)

To determine the role of c‐Cbl in thrombopoiesis, we generated c‐Cbl^fl/fl^Pf4^Cre^ mice specifically lacking c‐Cbl in the MK lineage (Figure 1A). While isolated B cells exhibited normal c‐CBL expression, knockout of c‐CBL protein in MKs and platelets (Plts) was confirmed by Western Blot (Figure 1B). Whole blood analyses revealed increased white blood counts (WBC) caused by a lymphocytosis and elevated numbers of small PLTs leading to a microthrombocytosis in c‐Cbl^fl/fl^Pf4^Cre^ mice, whereas red blood cells (RBC) were not altered (Figure 1C, Table 1 and Figure S2). To investigate the lymphocytosis of c‐Cbl^fl/fl^Pf4^Cre^ mice, we performed flow cytometry analyses with the peripheral blood and observed elevated B cell populations, mainly CD5^−^ B2 B cells, whereas T cells, NK cells and granulocytes were not altered (Figure 1D,E). Of note, leucocyte populations in the spleen, peritoneum and lymph nodes were not altered (Figure S3).

**TABLE 1:** Complete blood counts in c‐Cbl^fl/fl^ and c‐Cbl^fl/fl^Pf4^Cre^ mice

	c‐Cbl^fl/fl^	c‐Cbl^fl/fl^Pf4^cre^	*P*‐value
Mice	66	65	
Blood count (cells/µL)			
WBC (×10^3^)	9.6 ± 0.4	12.1 ± 0.5	.0002^a^
RBC (×10^6^)	10.3 ± 0.1	10.3 ± 0.1	.93^b^
Lymphocytes (×10^3^)	8.6 ± 0.4	11.0 ± 0.5	<.0001 ^a^
PLTs (×10^6^)	1.3 ± 0.4	1.9 ± 0.4	<.0001^b^
Monocytes (×10^2^)	2.7 ± 0.4	3.5 ± 0.5	.34^b^
Neutrophils (×10^2^)	4.0 ± 0.3	4.4 ± 0.3	.26^b^
Eosinophils (×10^2^)	1.9 ± 0.1	2.2 ± 0.1	.10^b^
MPV (fL)	7.0 ± 0.1	6.6 ± 0.1	.0062^a^
HGB (g/dL)	2.5 ± 0.1	2.5 ± 0.1	.67^b^

Abbreviations: HGB, haemoglobin; MPV, mean platelet volume; PLT, platelets; RBC, red blood count; WBC, white blood count.

^a^ Statistical analysis with Student's *t* test.

^b^ Statistical analysis with Mann‐Whitney test.

### 3.2 Megakaryopoiesis is increased in c‐Cbl^fl/fl^Pf4^Cre^ mice

**FIGURE 2:**
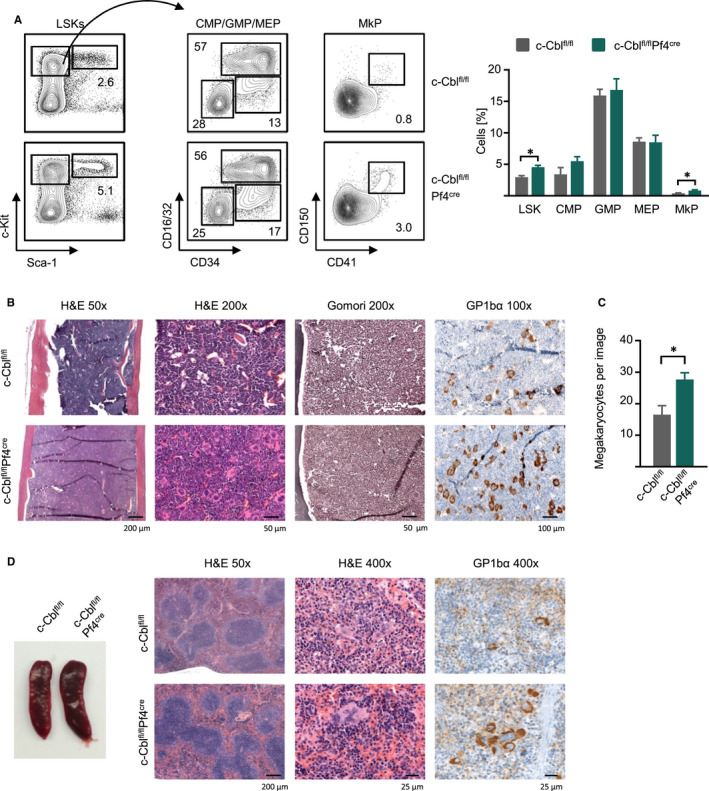
C‐Cbl‐deficient mice showed increased megakaryopoiesis. A, Representative FACS blots for flow cytometric analysis of LSKs (Lin^–^Sca‐1^+^c‐kit^+^), CMPs (Lin^–^Sca‐1^−^c‐kit^+^CD34^−^CD16/32^−^), GMPs (Lin^–^Sca‐1^−^c‐kit^+^CD34^+^CD16/32^−^), MEPs (Lin^–^Sca‐1^−^c‐kit^+^CD34^+^CD16/32^+^) and MkP (Lin^–^Sca‐1^−^c‐kit^+^CD41^+^CD150^+^) in the bone marrow and combined data of c‐Cbl^fl/fl^ and c‐Cbl^fl/fl^Pf4^cre^ mice at an age of 12‐16 wk (n = 4 per group, Mean ± SEM, *P ≤ .05). B, H&E staining, Gomori staining and immunohistochemistry for GPIbα of paraffin‐embedded bone marrow sections of one representative c‐Cbl^fl/fl^ and c‐Cbl^fl/fl^Pf4^cre^ mouse (age: 16‐18 wk). C, Number of GPIbα^+^ megakaryocytes per image (n = 2) identified by immunohistochemistry of paraffin‐embedded bone marrow sections of c‐Cbl^fl/fl^ and c‐Cbl^fl/fl^Pf4^cre^ mice (age: 16‐18 wk) (n = 3 per group, *P ≤ .05). D, A representative picture of spleens from c‐Cbl^fl/fl^ and c‐Cbl^fl/fl^Pf4^cre^ mice (age: 30 wk) (left) and H&E staining and immunohistochemistry for GPIbα of paraffin‐embedded spleen sections of one representative c‐Cbl^fl/fl^ and c‐Cbl^fl/fl^Pf4^cre^ mouse (age: 16‐18 wk) (right)

Next, we set out to characterize the role of c‐CBL in megakaryopoiesis and analysed hematopoiesis in the bone marrow of WT and c‐Cbl^fl/fl^Pf4^Cre^ mice. Flow cytometry analyses revealed increased Lin^−^Sca1^+^Kit^+^ (LSK) populations and enhanced megakaryocyte progenitors (MkPs) in c‐CBL‐deficient mice, while common myeloid progenitors (CMPs), granulocyte macrophage progenitors (GMPs) and MK and erythrocyte progenitors (MEPs) were not altered (Figure 2A). H&E stainings of bone marrow sections displayed normal cellularity of the marrow, and Gomori staining showed no signs of bone marrow fibrosis (Figure 2B). Detection of MKs by immunohistochemistry for GP1bα resulted in higher numbers of MKs in the marrow of c‐Cbl^fl/fl^Pf4^Cre^ mice compared to WT controls (Figure 2B,C). H&E and GPIbα staining of the WT spleen revealed a normal population of MKs, which were mostly located subcapsularly. In contrast, an increased number of MKs but only none to mild splenomegaly was observed in the spleen of c‐Cbl^fl/fl^Pf4^Cre^ animals. The MKs in the c‐Cbl^fl/fl^Pf4^Cre^ animals were slightly larger and hyperlobated (Figures 2D and S4).

### 3.3 c‐Cbl^fl/fl^Pf4^Cre^ mice exhibit increased thrombopoiesis

**FIGURE 3:**
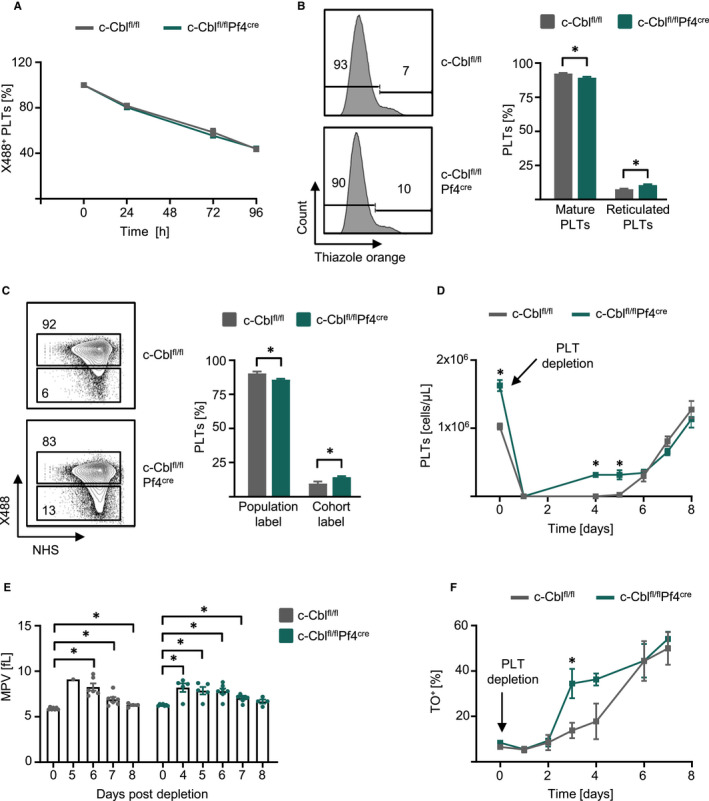
c‐Cbl^fl/fl^Pf4^Cre^ mice showed increased platelet recovery. A, c‐Cbl^fl/fl^ and c‐Cbl^fl/fl^Pf4^Cre^ mice (n = 4 per group, 10‐14 wk old) were iv injected with X488 (2 µg) antibody, and platelets lifespan was assessed by flow cytometry at the indicated time‐points (Mean ± SEM). B, RNA of platelets of c‐Cbl^fl/fl^ and c‐Cbl^fl/fl^Pf4^Cre^ mice (n = 6, 10‐14 wk old) was analysed with thiazole orange (TO) staining by flow cytometry. Representative plots of mature TO^−^ platelets and reticulated TO^+^ platelets (left) and combined results (right) (n = 10 mice per group, Mean ± SEM, *P ≤ .05). C, To determine the turnover of platelets, c‐Cbl^fl/fl^ and c‐Cbl^fl/fl^Pf4^Cre^ mice were iv injected with X488 (0.15 µg/g bodyweight) antibody and 24 h later iv injected with NHS‐biotin (600 µg). Platelets were analysed for double labelling (population label) and NHS single labelling (cohort label) after 3 h by flow cytometry (n = 3‐4 mice per group, 10‐14 wk old) (Mean ± SEM, *P ≤ .05). D, c‐Cbl^fl/fl^ and c‐Cbl^fl/fl^Pf4^Cre^ mice were analysed for initial platelet counts in the peripheral blood followed by platelet depletion with i.p. injection of R300 (3 µg/g bodyweight) antibody. Platelet recovery was monitored for 8 d. Combined data of three independent experiments are shown (n = 3‐7 mice per group, 10‐14 wk old) (Mean ± SEM, *P ≤ .05). E, MPV of platelets before (n = 7 per group) and after the depletion were analysed (Mean ± SEM, *P ≤ .05). F, c‐Cbl^fl/fl^ and c‐Cbl^fl/fl^Pf4^Cre^ mice were analysed for reticulated platelet by thiazole orange staining by flow cytometry followed by platelet depletion with i.p. injection of R300 (3 µg/g bodyweight) antibody. Reticulated platelet recovery was monitored for 7 d (n = 3 mice per group, 10‐14 wk old) (Mean ± SEM, *P ≤ .05)

After assessment of megakaryopoiesis, we further characterized the role of c‐CBL in platelet formation. In vivo labelling of platelets with an anti‐GPIbβ (X488) Ab displayed a comparable platelet lifespan in WT and c‐Cbl^fl/fl^Pf4^Cre^ mice, objecting prolonged survival of c‐Cbl‐deficient platelets (Figure 3A). However, intracellular RNA staining with TO resulted in increased percentage of reticulated platelets in the peripheral blood of c‐Cbl^fl/fl^Pf4^Cre^ mice (Figure 3B). In line, in vivo labelling of platelets with X488 and counterstaining with NHS after 24 hours demonstrated a higher platelet turnover in c‐Cbl^fl/fl^Pf4^Cre^ mice (Figure 3C).

To investigate whether platelet formation is not only affected in steady‐state conditions, we depleted platelets in vivo and monitored platelet recovery for 8 days. Again, c‐Cbl^fl/fl^Pf4^Cre^ mice showed increased platelet counts in the peripheral blood prior to the depletion and platelet recovery was significantly increased in c‐Cbl^fl/fl^Pf4^Cre^ mice compared to WT mice after 4‐5 days (Figure 3D). Furthermore, after depletion the mean platelet volume (MPV) of the recovered platelets increased; however, there was no significant difference between WT and c‐Cbl^fl/fl^Pf4^Cre^ mice (Figure 3E). Enhanced recovery of was due to enhanced regeneration and formation of c‐Cbl KO platelets which could be proved by increased reticulated TO^+^ platelets after the depletion and during the recovery phase within 2‐8 days in vivo (Figure 3F).

### 3.4 Impaired c‐MPL internalization in c‐Cbl^fl/fl^Pf4^Cre^ mice

**FIGURE 4:**
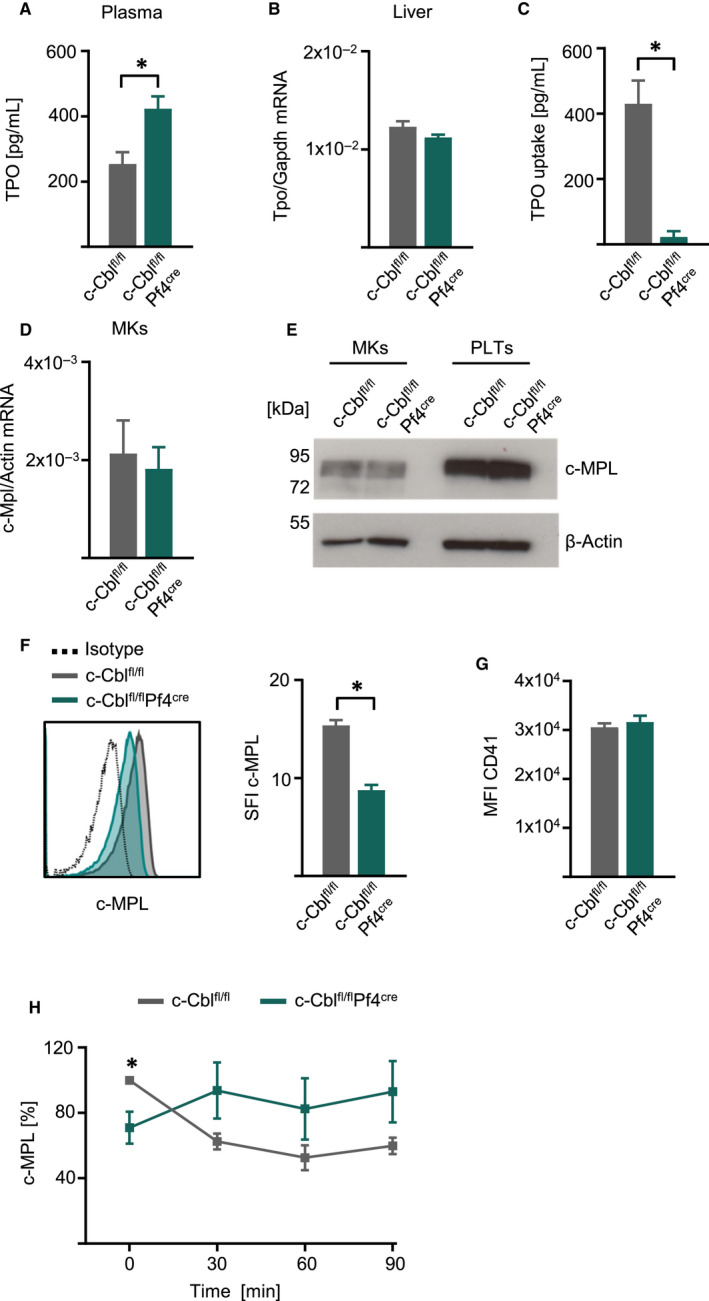
c‐Cbl^fl/fl^Pf4^Cre^ mice showed impaired TPO uptake and c‐Mpl internalization. A, Plasma of c‐Cbl^fl/fl^ and c‐Cbl^fl/fl^Pf4^Cre^ mice was harvested and TPO plasma levels were measured by ELISA (n = 10 mice per group, 10‐14 wk old) (Mean ± SEM, *P ≤ .05). B, TPO mRNA of livers from c‐Cbl^fl/fl^ and c‐Cbl^fl/fl^Pf4^Cre^ mice were analysed by qRT‐PCR. TPO gene expression was normalized to Gapdh (n = 5‐6 mice per group, 10‐14 wk old) (Mean ± SEM). C, To determine the TPO uptake platelets of c‐Cbl^fl/fl^ and c‐Cbl^fl/fl^Pf4^Cre^ mice were harvested and stimulated with TPO (2 ng/mL) for 2 h. TPO levels in the supernatant were measured by ELISA and the TPO uptake was calculated as follows $TPO\;pg/mL\;untreated\;control\;‐\;TPO\;pg/mL\;1\;\times \;10^{6}\;Platelets$ (n = 8 mice per group, 10‐12 wk old) (Mean ± SEM, *P ≤ .05). D, Megakaryocytes were generated from bone marrow of c‐Cbl^fl/fl^ and c‐Cbl^fl/fl^Pf4^Cre^ mice. C‐Mpl mRNA level were measured by qRT‐PCR and normalized to Actin expression (n = 5‐6 per group, 12‐16 wk old) (Mean ± SEM). E, Megakaryocytes were generated from bone marrow of c‐Cbl^fl/fl^ and c‐Cbl^fl/fl^Pf4^Cre^ mice and platelets were harvested. Expression of c‐MPL was assessed by Western blot analysis. One exemplary result with β‐Actin as loading control is shown. F, Platelets of c‐Cbl^fl/fl^ and c‐Cbl^fl/fl^Pf4^Cre^ were stained for c‐MPL surface expression. SFIs were calculated with respective isotype controls (n = 3‐4 per group, 10‐14 wk old) (Mean ± SEM, *P ≤ .05). G, Platelets of c‐Cbl^fl/fl^ and c‐Cbl^fl/fl^Pf4^Cre^ were stained for CD41 surface expression (n = 4 per group, 10‐14 wk old) (Mean ± SEM). H, Platelets of c‐Cbl^fl/fl^ and c‐Cbl^fl/fl^Pf4^Cre^ mice were isolated and treated with TPO (25 ng/mL) for the indicated time‐points. C‐MPL internalization was measured by flow cytometry. Combined data of three independent experiments is shown (n = 3‐5 per group, 10‐14 wk old) (Mean ± SEM, *P ≤ .05)

Since the ubiquitin ligase c‐CBL is involved in ubiquitination of the TPO receptor c‐MPL and thereby in negatively regulating TPO signalling, we aimed to determine TPO signalling in WT and c‐Cbl^fl/fl^Pf4^Cre^ platelets. First, TPO plasma levels were significantly enhanced in c‐Cbl^fl/fl^Pf4^Cre^ mice (Figure 4A). Interestingly, TPO mRNA levels in the liver were comparable between WT and c‐Cbl^fl/fl^Pf4^Cre^ mice, indicating that elevated TPO plasma levels were not caused by enhanced TPO synthesis (Figure 4B). Of note, plasma levels of other plasma components were similar in both cohorts, except of non‐esterified fatty acids which were elevated in c‐Cbl^fl/fl^Pf4^Cre^ mice (Table 2). Next, we determined the capacity of platelets to take up TPO and therefore incubated isolated platelets of WT and c‐Cbl^fl/fl^Pf4^Cre^ mice with recombinant TPO and measured TPO levels in the supernatant after 2 hours. CBL‐deficient platelets showed a severe decrease in TPO uptake capacity compared to WT platelets (Figure 4C).

**TABLE 2:** Plasma analysis of c‐Cbl^fl/fl^ and c‐Cbl^fl/fl^Pf4^Cre^ mice

	c‐Cbl^fl/fl^	c‐Cbl^fl/fl^Pf4^cre^	*P*‐value
Mice	9	9	
Plasma analysis			
Urea (mg/dL)	50.2 ± 1.5	51.1 ± 2.2	.74
Uric acid (mg/dL)	2.0 ± 0.3	2.3 ± 0.3	.49
Bilirubin (mg/dL)	0.4 ± 0.0	0.4 ± 0.0	1.00
Total protein (g/dL)	5.1 ± 0.1	4.9 ± 0.1	.35
Cholesterol (mg/dL)	84.4 ± 5.3	83.1 ± 4.8	.85
HDL Cholesterol (mg/dL)	24.0 ± 2.4	24.4 ± 1.9	.88
LDL Cholesterol (mg/dL)	4.0 ± 0.0	4.4 ± 0.4	.33
Triglyceride (mg/dL)	61.3 ± 3.8	64.9 ± 5.8	.61
Non‐esterified fatty acids (µmol/L)	895 ± 29	1189 ± 115	.0371^a^
Creatine kinase (U/L)	349 ± 89	404 ± 120	.71
Glutamate oxalacetal transaminase (U/L)	91 ± 8.5	92 ± 9.7	.91
Glutamate pyruvate transaminase (U/L)	58 ± 6.9	49 ± 2.4	.24
Alkaline phosphatase (U/L)	17 ± 3.4	17 ± 2.9	1.00

^a^ Statistical analysis with Student's *t* test.

One explanation would be decreased c‐MPL expression which could lead to impaired TPO uptake in CBL‐deficient platelets. However, qRT‐PCR and Western blot analysis of total c‐MPL expression in MKs and c‐MPL protein levels in platelets were comparable in WT and c‐Cbl^fl/fl^Pf4^Cre^ mice (Figure 4D,E). We further analysed c‐MPL surface expression by flow cytometry and detected distinct lower levels of c‐MPL surface expression on platelets of c‐Cbl^fl/fl^Pf4^Cre^ mice, while other platelets receptors like CD41 were not affected (Figure 4F,G).

Finally, we performed in vitro c‐MPL receptor internalization analyses and determined c‐MPL surface expression after TPO stimulation. In WT platelets stimulation with TPO led to internalization of ~50% the c‐MPL receptor within 60 minutes, in contrast c‐Cbl^fl/fl^Pf4^Cre^ platelets showed severe defects in receptor internalization (Figure 4H).

### 3.5 c‐Cbl^fl/fl^Pf4^Cre^ mice showed constitutive active c‐MPL signalling

**FIGURE 5:**
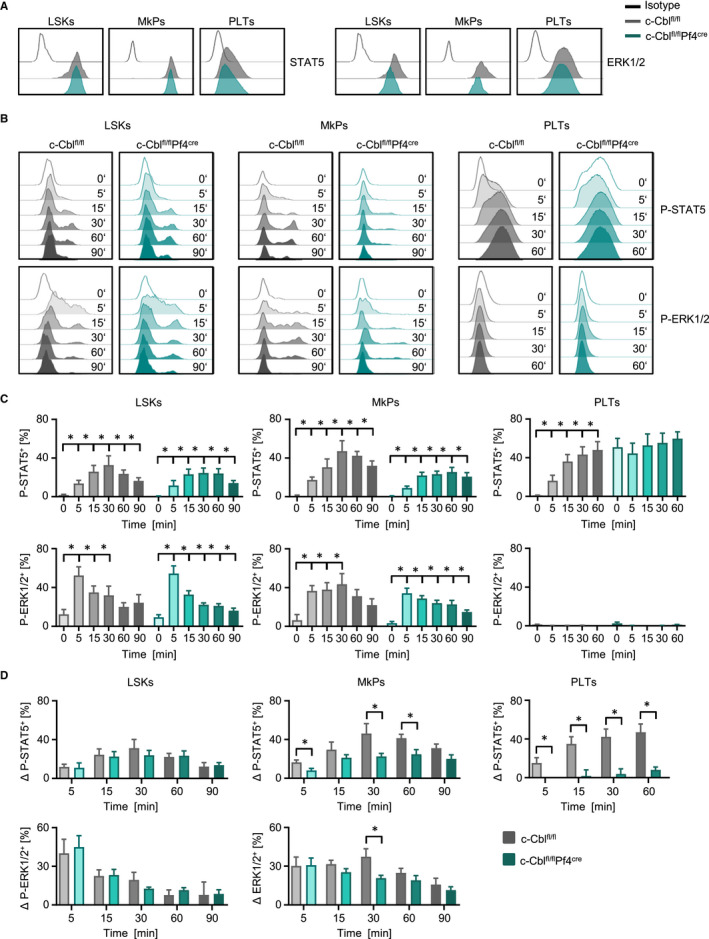
PLTs of c‐Cbl^fl/fl^Pf4^Cre^ mice showed impaired c‐MPL receptor signalling. After staining of bone marrow cells and PLTs cells were stimulated with 100 ng/mL TPO for the indicated time‐points and c‐Mpl receptor activation was assessed by intracellular staining of STAT5/P‐STAT5(Y694) and ERK1/2/P‐ERK1/2(T202/Y204) in LSKs (Lin^–^Sca‐1^+^c‐kit^+^), MkP (Lin^–^Sca‐1^−^c‐kit^+^CD41^+^CD150^+^) and PLTs (CD41^+^) with flow cytometry. A, Total STAT5 and ERK protein expression in LSKs, MkPs and PLTs of one representative result out of three independent experiments is shown. B, Exemplary results for P‐STAT5 and P‐ERK induction after TPO stimulation of LSKs, MkPs and PLTs for one representative mouse per genotype (12‐16 wk old) are shown. C, Pooled result of P‐STAT5^+^ and P‐ERK1/2^+^ for LSKs (n = 6‐8 per group), MkPs (n = 5‐7) and PLTs (n = 5‐8 per group) at the indicated time‐points of is shown (Mean ± SEM, *P ≤ .05). D, Induction of P‐STAT5^+^ and P‐ERK1/2^+^ in LSKs, MkPs and PLTs was calculated by “% positive cells at x min” − “% positive cells at 0 min” (Mean ± SEM, *P ≤ .05)

To further elucidate the impact of c‐Cbl deletion on the regulation of c‐MPL signalling, we stimulated isolated bone marrow cells and platelets from WT and c‐Cbl^fl/fl^Pf4^Cre^ mice with TPO and analysed receptor activation via STAT5 and ERK1/2 phosphorylation. While total protein expression of STAT5 and ERK1/2 was comparable in LSKs, MkPs and PLTs of WT and c‐Cbl^fl/fl^Pf4^Cre^ mice (Figure 5A), stimulation of the c‐MPL receptor showed induction of P‐STAT5 and P‐ERK (Figure 5B). Maximum phosphorylation of STAT5 and ERK1/2 in LSKs and MkPs was observed after ~30 minutes for STAT5 and after ~5 minutes of TPO stimulation for ERK1/2 (Figure 5C,D). Whereas c‐MPL of LSKs showed comparable signalling capacity in WT and c‐Cbl^fl/fl^Pf4^Cre^ mice, MkPs of c‐Cbl^fl/fl^Pf4^Cre^ mice displayed impaired phosphorylation levels of STAT5 and ERK1/2 (Figure 5D). Interestingly, while TPO stimulation of WT mice showed expected induction of P‐STAT5 over time, STAT5 signalling in PLTs of c‐Cbl^fl/fl^Pf4^Cre^ mice was constitutive active and TPO could not induce further activation (Figure 5C). In general, TPO stimulation of PLTs did not induce any P‐ERK1/2 at all. Of note, phosphorylation of ERK1/2 could be achieved by stimulation of PLTs with PMA/Ionomycin, proving ERK1/2 signalling capacity in general (Figure S5).

## 4 DISCUSSION

In this study, we investigated the role of the E3 ubiquitin ligase c‐Cbl in the MK linage. Known mutations in the Cbl gene are nucleotide substitutions or small insertions/deletions in exons 8 and 9 of the gene which lead to modification of the linker or the RING finger domain.^17^ These mutations are also found in rare cases of myeloproliferative disorders like ET and primary myelofibrosis and can result in a loss of function of the ubiquitin E3 ligase activity, thereby disrupting the ubiquitin/proteasome‐mediated degradation of MPL/JAK2.^18^ Moreover, in a recent human genome‐wide association study, single nucleotide polymorphisms in or near the c‐Cbl gene was associated with observed platelet counts.^19^

Our data show that c‐Cbl plays a key role in negative feedback regulation of TPO signalling via its receptor c‐MPL, which is critical for the maintenance of hematopoietic stem cells in addition to being the key regulator of megakaryopoiesis.^20^ Our previous work has identified c‐Cbl as E3 ubiquitin ligase involved in the ubiquitination and degradation of c‐MPL as well as that knock out of c‐Cbl in a cell‐based model caused a hyperproliferative phenotype.^9^ Recently, this negative regulatory role of c‐Cbl in downmodulation of c‐MPL expression was further supported in a mouse model of ABCG4 knockout mice.^21^ C‐Cbl null mice showed a severe thrombocytosis compared to WT mice, while there was no difference in total leucocyte count. In addition, c‐Cbl null mice developed a marked splenomegaly with elevated number of MK, while there was no difference in bone marrow cellularity.^22^ In contrast to these findings, our c‐Cbl^fl/fl^Pf4^Cre^ mice showed lymphocytosis but no noteworthy splenomegaly.

The thrombocytosis observed in the c‐Cbl^fl/fl^Pf4^Cre^ mice was most likely due to elevated numbers of bone marrow MKs, MkPs and spenic MK compared to WT mice since the half lifespan of the platelets was not affected. This theory is supported by the faster platelet recovery observed in c‐Cbl^fl/fl^Pf4^Cre^ mice after platelet depletion.

Regarding the alterations observed in LSKs and B cell populations, we suggest different hypotheses. C‐Cbl deficiency was achieved by deletion of a PF4‐induced Cre recombinase, which was previously shown to be already expressed in LSK stem cells and lymphoid progenitors, which partially led to expression in B cells.^23^, ^24^ Furthermore, it is known that TPO/c‐MPL signalling plays an important role in maintaining HSCs during adult hematopoiesis since TPO^−/−^ showed an age‐progressive loss of HSCs and reduced MkPs.^25^ This could be one explanation how c‐Cbl KO mice displaying elevated TPO levels also exhibit increased LSK stem cells and MkPs. Elevated LSKs levels could probably lead to elevated B cells in the periphery; however, in our view there is a second explanation. Au and coworkers characterized an altered B lymphopoiesis caused by deregulated TPO signalling.^26^ They could show that enhanced TPO signalling in TPO^Tg^ mice led to increased number of splenocytes. We did not analyse TPO signalling in B cells of our c‐Cbl^fl/fl^Pf4^Cre^, but one could speculate that residual PF4‐cre activity in B cells might influence TPO signalling and thereby could lead to elevated B cell counts. A third theory independent of altered B cell c‐MPL signalling or PF4 expression may simply account the inability of TPO uptake of c‐Cbl KO platelets. This was causing elevated TPO levels and thereby influencing B cell development and leading to increased B2 B cells which are the main splenic B cell population usually consisting of transitional, marginal zone and follicular B cells.

C‐MPL internalization/endocytosis was severely impaired in c‐Cbl^fl/fl^Pf4^Cre^ platelets, resulting in constitutive STAT5 phosphorylation and elevated circulating TPO levels. TPO is the main cytokine regulating megakaryopoiesis and its receptor c‐MPL, signals through JAK2 phosphorylation and activation, followed by phosphorylation and nuclear translocation of signal transducers and activators of transcription.^27^ Subsequently, c‐MPL is down‐regulated by clathrin‐dependent endocytosis.^20^, ^28^, ^29^ TPO‐independent c‐MPL signalling as observed in c‐Cbl^fl/fl^Pf4^Cre^ platelets is consistent with findings of others describing constitutive signalling due to impaired receptor‐mediated endocytosis in cells lacking DNM2 and DNM2‐null platelets.^30^, ^31^, ^32^, ^33^

In contrast, LSKs did not show any significant differences in c‐MPL signalling, while c‐Cbl‐deficient MkPs displayed a decreased P‐STAT5 and P‐ERK1/2 induction after TPO stimulation. Hypotheses that c‐MPL signalling could eventually be regulated or silenced by other ubiquitin ligases like Cbl‐b or potentially compensated by different signalling pathways especially in multipotent progenitor cells could serve to explain our data. Final clarification warrants further analyses.

Internalization of TPO after binding and stimulation to c‐MPL is an important method for regulating plasma TPO levels.^33^, ^34^, ^35^, ^36^ Interestingly, the R102P c‐MPL mutation is known to lead to a loss of function of the c‐MPL receptor, due to blocked processing in the endoplasmic reticulum and lack of c‐MPL surface expression, causing congenital amegakaryocytic thrombocytopenia (CAMT). Heterozygous mutations cause a paradoxical thrombocytosis characterized by high TPO level in plasma due to incomplete cell surface expression of c‐MPL in mature cells and increased stimulation of Stem/progenitors population with optimal expression of c‐MPL. CAMT patients and our c‐Cbl^fl/fl^Pf4^Cre^ mice have elevated circulating TPO levels.^37^ In contrast, the P106L MPL mutation leads to a hereditary thrombocytosis. P106L mutated MPL receptor has a reduced capability to bind TPO leading to an internalization defect causing supernatural TPO levels in the serum of these patients. TPO‐induced c‐MPL signalling was also impaired in the mutant form of the receptor.^38^ Platelets of c‐Cbl^fl/fl^Pf4^Cre^ mice show reduced expression of c‐MPL on the cell surface, while total protein was similar compared to WT mice. Elevated TPO levels observed in c‐Cbl^fl/fl^Pf4^Cre^ mice could be explained by a defect in c‐MPL trafficking and internalization. This is similar to the c‐MPL internalization defected observed in Dnm2^fl/fl^Pf4^Cre^ mice and thereby causing elevated TPO serum levels.^39^ C‐Mpl^fl/fl^Pf4^Cre^ mice develop a myeloproliferative phenotype with an increased number of stem and progenitor cells and increased number of MK and platelets. Other than expected, these mice show normal plasma TPO levels. An explanation could be the expansion of c‐MPL‐expressing stem/progenitor cells thereby normalizing the serum TPO concentration.^40^

In contrast to the autoregulation model of sera TPO, these levels are lower than expected in patients with immune thrombocytopenia ^35^, ^41^ and higher in patients with ET.^42^

Our data support a model that such disorders may be, in part, underpinned by insufficient or dysfunctional c‐MPL mass within the platelet/MK pool, resulting in increased TPO stimulation of the c‐MPL expressing stem and progenitor cells, similar to what was observed in c‐MPL^fl/fl^Pf4^Cre^, DNM2^fl/fl^Pf4^Cre^, Jak2^fl/fl^Pf4^Cre^ and c‐Cbl^fl/fl^Pf4^Cre^ mice.

## CONFLICT OF INTEREST

The authors confirm that there are no conflicts of interest.

## AUTHOR CONTRIBUTIONS

**Melanie Märklin:** Conceptualization (equal); Data curation (lead); Formal analysis (lead); Funding acquisition (supporting); Investigation (equal); Methodology (lead); Supervision (equal); Validation (lead); Visualization (lead); Writing‐original draft (lead); Writing‐review & editing (equal). **Claudia Tandler:** Investigation (equal); Methodology (equal); Writing‐review & editing (equal). **Hans‐Georg Kopp:** Conceptualization (equal); Project administration (supporting); Supervision (equal); Writing‐original draft (supporting); Writing‐review & editing (supporting). **Kyle L Hoehn:** Methodology (supporting); Validation (equal); Writing‐original draft (supporting); Writing‐review & editing (supporting). **Leticia Quintanilla‐Martinez:** Formal analysis (equal); Validation (equal); Visualization (equal); Writing‐review & editing (supporting). **Oliver Borst:** Formal analysis (equal); Methodology (equal); Validation (equal); Writing‐original draft (equal); Writing‐review & editing (equal). **Martin R. Müller:** Conceptualization (equal); Funding acquisition (lead); Project administration (equal); Supervision (equal); Writing‐original draft (equal); Writing‐review & editing (equal). **Sebastian J. Saur:** Conceptualization (lead); Funding acquisition (lead); Methodology (equal); Project administration (equal); Supervision (lead); Validation (equal); Visualization (equal); Writing‐original draft (lead); Writing‐review & editing (lead).

## Supporting information

Fig S1‐S5

## Data Availability

The data sets used and/or analysed during the current study are available from the corresponding author on reasonable request.
