# Supplementary material for: C‐Cbl regulates c‐MPL receptor trafficking and its internalization
Source: J Cell Mol Med. 2020 Sep 20;24(21):12491–503. doi: 10.1111/jcmm.15785 (PMC7687000; doi:10.1111/jcmm.15785)
Supplement: Supplementary file 1 — Fig S1‐S5 [file JCMM-24-12491-s001.pdf]

Supplementary File

C-Cbl Regulates c-MPL Receptor Trafficking and its Internalization

Melanie Märklin[1] (PhD), Claudia Tandler (M.Sc)[1], Hans-Georg Kopp[3] (MD), Kyle L. Hoehn[4] (PhD), Leticia Quintanilla-Martinez[5] (MD), Oliver Borst[6] (MD), Martin R. Müller[2,7*] (MD, PhD) and Sebastian J. Saur[2] (MD)

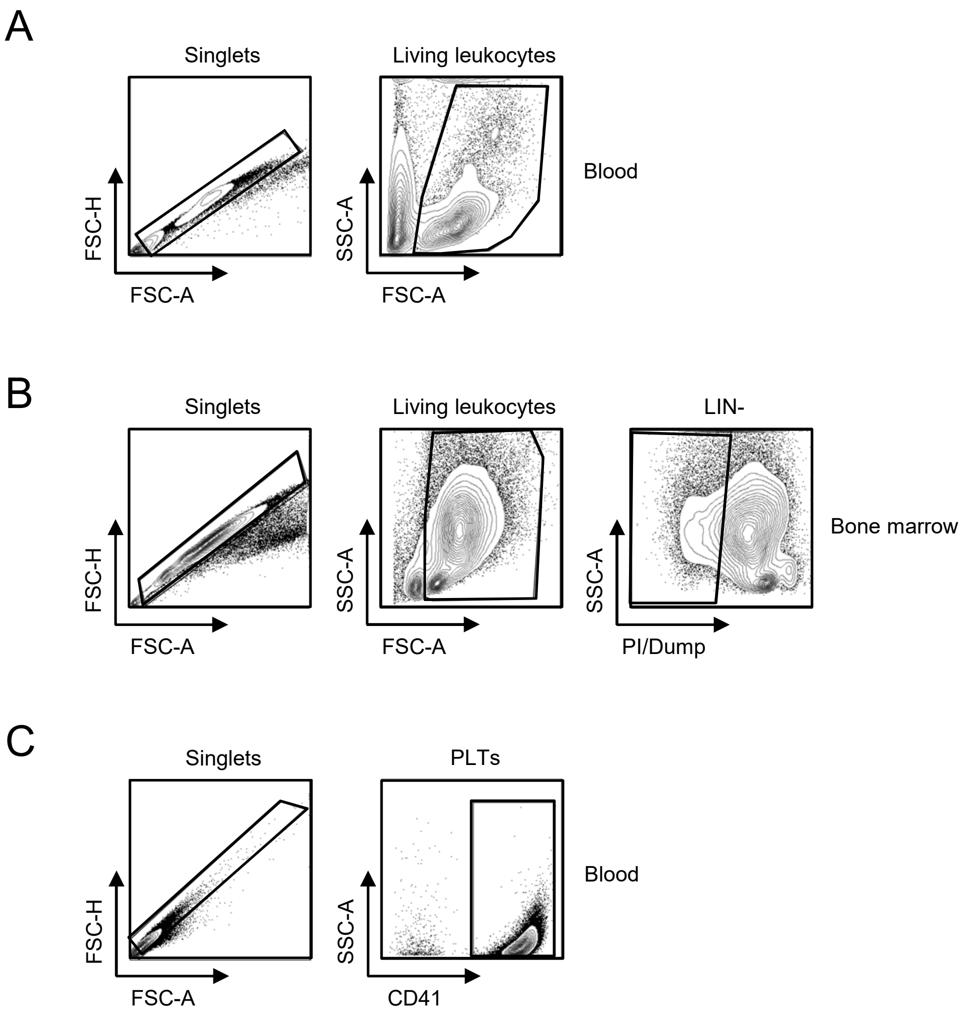

Supplementary Figure S1: Gating strategy for flow cytometry analyses

(A) Exemplary hierarchical gating of singlets and living leucocytes in the peripheral blood.

(B) Exemplary hierarchical gating of singlets, living leucocytes and LIN^- cells in the bone marrow. LIN^- was considered as propidium iodide (PI) negative and B220/CD11b/CD3/Ter119/Gr-1/CD127 negativity.

(C) Exemplary hierarchical gating of singlets and living $CD41^+$ platelets.

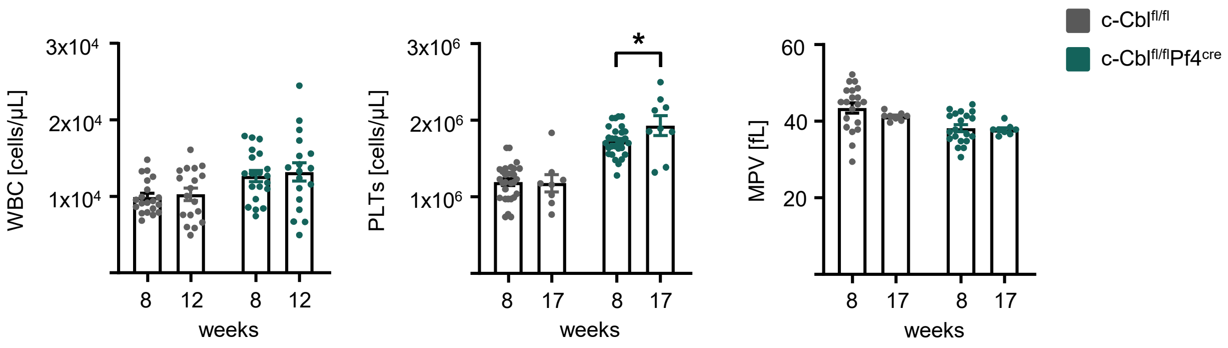

Supplementary Figure S2: Blood analysis at different timepoints

White blood count (WBC), platelet numbers (PLTs) and mean platelet volume (MPV) in c-Cbl$^{fl/fl}$ and c-Cbl$^{fl/fl}$Pf4^{Cre} mice were analyzed at a different age (Mean ± SEM, * $p \leq 0.05$).

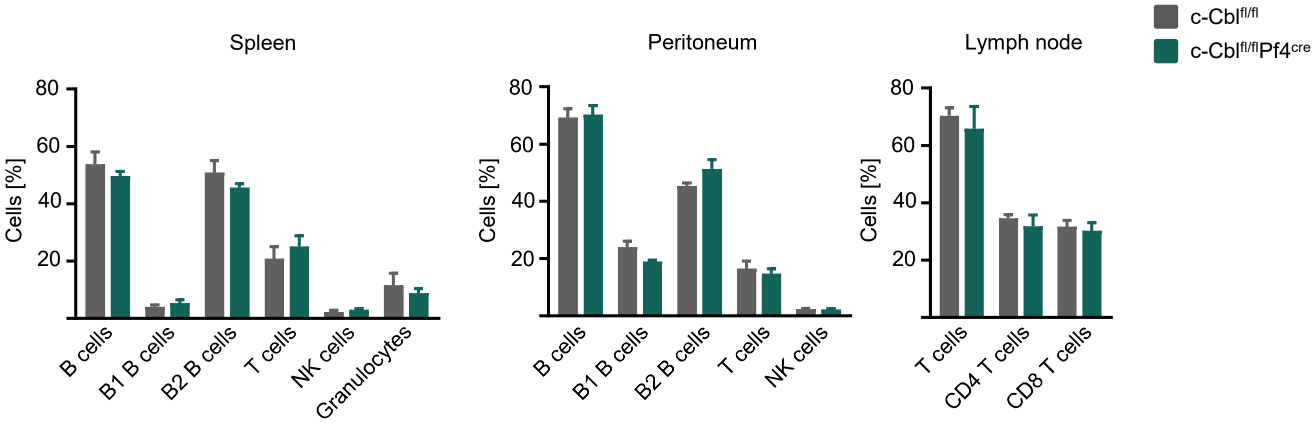

Supplementary Figure S3: Leukocyte analysis of different organs

(A) Flow cytometric analysis of the spleen, peritoneum and the lymph nodes of c-Cbl$^{fl/fl}$ and c-Cbl$^{fl/fl}$Pf4^{Cre} (n=4 per group, Mean ± SEM).

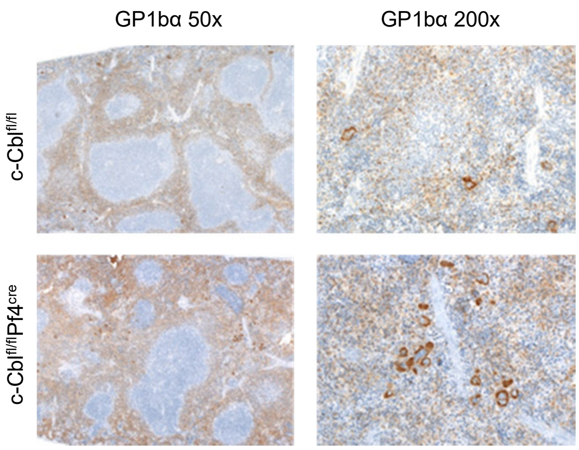

Supplementary Figure S4: GPIbα staining in the spleen

Immunohistochemistry for GPIbα of paraffin-embedded spleen sections of one representative c-Cbl$^{fl/fl}$ and c-Cbl$^{fl/fl}$Pf4^{Cre} mouse in different magnifications.

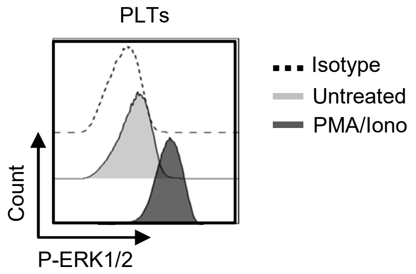

Supplementary Figure S5: P-ERK induction in PLTs with PMA/Iono
PLTs cells were stimulated with PMA (50 ng/mL) + Ionomycin (500 ng/mL) for 10 min and intracellular staining of P-ERK1/2(T202/Y204) was assessed with flow cytometry.
